# Antibodies directed towards neuraminidase restrict influenza virus replication in primary human bronchial epithelial cells

**DOI:** 10.1371/journal.pone.0262873

**Published:** 2022-01-31

**Authors:** Anouk Smet, Joao Paulo Portela Catani, Tine Ysenbaert, Amanda Gonçalves, Harry Kleanthous, Thorsten U. Vogel, Xavier Saelens, Emma R. Job

**Affiliations:** 1 VIB-UGent Medical Biotechnology Centre, VIB, Ghent, Belgium; 2 Department of Biomedical Molecular Biology, Ghent University, Ghent, Belgium; 3 Department of Biochemistry and Microbiology, Ghent University, Ghent, Belgium; 4 VIB BioImaging Core, Ghent, Belgium; 5 VIB-UGent Center for Inflammation Research, Ghent, Belgium; 6 Sanofi Pasteur, Research North America, Cambridge, Massachusetts, United States of America; Erasmus University Medical Center, NETHERLANDS

## Abstract

Influenza neuraminidase (NA) is implicated in various aspects of the virus replication cycle and therefore is an attractive target for vaccination and antiviral strategies. Here we investigated the potential for NA-specific antibodies to interfere with A(H1N1)pdm09 replication in primary human airway epithelial (HAE) cells. Mouse polyclonal anti-NA sera and a monoclonal antibody could block initial viral entry into HAE cells as well as egress from the cell surface. NA-specific polyclonal serum also reduced virus replication across multiple rounds of infection. Restriction of virus entry correlated with the ability of the serum or monoclonal antibody to mediate neuraminidase inhibition (NI). Finally, human sera with NI activity against the N1 of A(H1N1)pdm09 could decrease H6N1 virus infection of HAE cells, highlighting the potential contribution of anti-NA antibodies in the control of influenza virus infection in humans.

## Introduction

Influenza viruses pose a serious public health threat. Influenza A and B viruses carry two major surface glycoproteins: hemagglutinin (HA) and neuraminidase (NA). The HA binds to sialic acid present on the surface of target cells. The NA has an opposing function, cleaving the linkage between the sialic acid and the adjacent sugar residue [1]. NA activity aids in virion entry into underlying cells by cleaving sialic acid residues from decoy receptors in the airways [2, 3]. In concert with HA, NA may also allow the influenza virion to reach a suitable endocytic patch on the cell surface by promoting a grasping and rolling mechanism [4, 5]. Furthermore, NA activity has been shown to contribute to HA-mediated membrane fusion based on the use of HA pseudotyped lentiviral reporter viruses [6]. At a late stage in the influenza virus replication cycle, during virion budding, NA activity facilitates the release of newly produced virions from the cell surface and prevents virion self-aggregation [7].

Preclinical and clinical studies have demonstrated that antibodies that can block NA activity contribute to protection against influenza. Vaccination with recombinant NA, for example, can protect mice against a challenge with an otherwise lethal dose of homologous virus and this protection has been shown to correlate with the levels of NA-inhibiting antibodies [8, 9]. Furthermore, administration of monoclonal antibodies with neuraminidase inhibition (NI) activity fully protected mice from disease when challenged with influenza virus [10–13]. NA-specific monoclonal antibodies without detectable NI, however, can also protect mice against influenza A virus challenge by a mechanism that relies on the engagement of Fcγ receptors [14–17]. Importantly, controlled human influenza virus challenge studies have demonstrated that a reduction in clinical symptoms correlated with the presence of NI antibodies [18, 19]. It is well accepted that antibodies with NI activity can reduce the spread of the virus from infected cells [20]. However, as NA plays several roles during the viral life cycle, it is likely that NA antibodies could interfere at any of these steps. Indeed, small molecule NA inhibitors such as oseltamivir and zanamivir prevent desialylation of innate soluble proteins by NA [3, 21] and decrease viral entry into human cell lines [3, 4].

Human airway epithelial (HAE) cells harvested at the intrathoracic portion of the trachea (distal trachea) and carina can form a pseudostratified epithelium consisting of tight junctions, basal cells, ciliated cells and mucus-producing goblet cells upon differentiation in air-liquid phase cultures [22]. Such differentiated HAE cultures represent the *in vitro* gold standard host cell model to examine human influenza virus infection and accurately reflect the receptor diversity found in humans [23]. Herein, we addressed the impact of NA targeting antibodies upon influenza A virus infection using differentiated primary HAE cells. We used monoclonal antibodies and polyclonal sera, directed to the NA of A(H1N1)pdm09 to investigate the possible role of NA antibodies during virus entry and replication in HAE cultures. Our data show that antibodies with NI activity can reduce initial infection and delay the release of nascent virus in HAE cells, highlighting the protective potential of anti-NA antibodies within the human airways.

## Methods

### Mouse and human ethical statement

All mouse experiments complied with national (Belgian Law 14/08/1986 and 22/12/20333, Belgian Royal Decree 06/04/2010) and European legislation (EU Directives 2010/63/EU, 86/609EEG) on animal regulations. The ethics committee of the VIB and Ghent University Faculty of Science (Eth. Com No. 2014–068 and 2016–059) approved all mouse experiments. Post-2009 human serum samples were collected under the ethical number EC UZG 20018/0380, as approved by the commission for medical ethics, University Hospital Gent.

### Viruses

We used a derivative of the H1N1 2009 pandemic virus strain A/Belgium/1/2009 (Bel/09) that had been mouse-adapted (maBel/09) and shows a more robust growth phenotype on Madin Darby canine kidney (MDCK) cells [24]. In addition, a 6:2 H6N1 reverse genetic (RG) strain carrying the HA of the H6N1 virus A/mallard/Sweden/81/2002, the NA of Bel/09 and the other 6 gene segments of A/PR/8/34 (PR8/34) was used in this study. Viruses were propagated in MDCK cells in serum-free medium and infectious virus titers were determined by plaque assay on MDCK cells under an 0.6% Avicel RC-591 overlay. Plaques were visualized by staining with polyclonal goat anti-influenza ribonucleoprotein (RNP) (1/3,000, obtained through the NIH Biodefense and Emerging Infections Research Resources Repository, NIAID, NIH: Polyclonal Anti-Influenza Virus RNP, A/Scotland/840/74 (H3N2), (antiserum, Goat), NR-3133)) followed by secondary anti-goat IgG HRP-linked antibody (GE Healthcare) [13]. A tissue culture infectious dose 50% (TCID_50_) assay was used to determine viral titers on supernatants from infected HAE cells. In brief, MDCK cells in 96-wells plate were inoculated with 10-fold serial dilutions of HAE cell culture supernatant in serum-free DMEM containing 1 μg/ml of TPCK-treated trypsin (Sigma Aldrich). Virus was detected after 7 days by agglutination of chicken or turkey red blood cells.

### Sera and monoclonal antibodies

One μg of recombinant soluble trimeric HA or tetrameric NA derived from the Bel/09 strain [24] was used to immunize BALB/c mice by subcutaneous injection with a prime-boost strategy in the presence of Sigma adjuvant system (SAS, Sigma Aldrich). Negative control sera were prepared from mice that had been mock-vaccinated with buffer alone (PBS) plus SAS. Three weeks after the boost vaccination blood was collected to prepare serum. Human sera were obtained from healthy volunteers 7 days following vaccination with a licensed 2017–2018 seasonal quadrivalent influenza vaccine. The full vaccination and infection history of the donors is unknown. Sera were heat-inactivated at 56°C for 1h and, for infection assays, also treated with the receptor-destroying enzyme from *Vibrio Cholera* (Sigma Aldrich), in accordance with the WHO protocol [25].

The N1-specific NA mouse monoclonal antibodies used were the previously described N1-C4 and N1-7D3 [13], and the anti-NA rabbit monoclonal antibody HCA-2 [26]. A mouse IgG1 monoclonal antibody directed against NBe of influenza B virus was used as an irrelevant control.

### HAE cells and influenza virus infection assay

HAE cells from healthy human donors were sourced from either Lonza or Epithelix, as specified in the figure legends. Lonza cells were differentiated and air-lifted in-house according to the manufacturer’s directions. The Epithelix MucilAir system was already airlifted and differentiated and thus ready for use. Differentiation was confirmed by visual inspection of the cells, including the presence of beating cilia and mucus production. The cells were incubated at 37°C with 5% CO_2_ in B-ALI^TM^ medium (Lonza) or MucilAir culture medium (Epithelix), with media refreshment in the basal chamber every 2–3 days.

For fluorescent focus reduction assays, HAE cells were pre-washed with cell culture media to remove the surplus amount of mucus that had accumulated over time on the apical side. This washing step did not decrease the polysaccharide and mucin layer associated with the HAE cells, as revealed by Periodic Acid-Schiff (PAS) staining (Sigma) (S1 Fig). HAE cultures were subsequently infected at a multiplicity of infection (MOI) of 1 or 0.1 (based on MDCK titration values) with the indicated viruses that had been pre-incubated for 30 minutes at 37°C with (i) mouse or human sera, (ii) NA-specific or isotype control monoclonal antibodies, (iii) Oseltamivir, or (iv) medium only. Subsequently, the pre-incubated viruses were allowed to bind to the cells for 1 h at the apical side of the HAE cells. The virus inoculum was subsequently washed away, and the cells were cultured for a further 7 or 23 hours in the air-liquid phase. Cells were then fixed with 4% paraformaldehyde (PFA) and permeabilized with 0.15% Triton X100. In some experiments, prior to fixation, the cells were washed once with culture media and the supernatant was kept for titration [13]. Following permeabilization, monolayers were blocked with 3% bovine serum albumin (BSA) and stained with a goat polyclonal anti-influenza virus RNP (NR-3133, BEI resources, NIAID, NIH) and Alexa Fluor 488-labelled donkey anti-goat IgG (Invitrogen). Hoechst co-stain was used to visualize the nucleus. Membranes were finally cut from their supports and mounted on glass slides for microscopy. Slides were imaged with a Zeiss 880 confocal microscope with 25x/0.8 numerical aperture multi-immersion objective and the analysis of images was performed with Fiji (NIH) software or with Volocity (Perkin Elmer).

For multi-step growth curves, virus inoculations were performed at MOIs of 0.1 or 0.01. Viruses were pre-incubated for 1 hour at 37°C with either (i) NA-specific or control sera, (ii) NA-specific or negative control monoclonal antibodies, (iii) Oseltamivir, or (iv) with medium, as indicated. Preincubated viruses were then added to pre-washed HAE cells for 1 hour at 37°C, after which time the virus inoculum was washed away. In some setups the infection was allowed to proceed in the air-liquid phase. At the indicated time-points virus was sampled from the apical side by washing with 240 μl of medium, to determine viral titers using TCID_50_. In other setups, at the indicated time-points, the anti-NA antibody/inhibitor/serum was re-added (in 50μl of medium) after sampling to maintain a constant concentration of antibody/inhibitor/serum.

For transmission electron microscopy (TEM) experiments the virus inoculum (MOI 1) was pre-incubated with mouse anti-NA serum (1/100 dilution), N1-C4 monoclonal antibody (10 μg/ml), Oseltamivir (24 μg/ml), or medium and added to HAE cells for 1 h. The inoculum was washed away and the treatment with anti-NA serum, N1-C4, Oseltamivir, or medium without virus was re-added to the cells. The infection was allowed to proceed for 16 h, at which point the cells were fixed with 4% PFA and 2.5% glutaraldehyde in 0.1 M cacodylate buffer pH 7,4 for 2.5 h at room temperature and then overnight at 4°C. Fixed membranes were removed from their inserts and prepared for TEM as described previously [27]. All sera used in HAE infection assays were heat-inactivated and RDE treated.

### Neuraminidase inhibition (NI) assay

NI assays were performed using the enzyme-linked lectin assay (ELLA) [28]. Serial dilutions, prepared in 2-(N-morpholino)ethanesulfonic acid (MES) pH 6.5 supplemented with 10 mg/ml BSA, 1 mM CaCl_2_, 0.5 mM MgCl_2_ and 0.5% Tween 20, of monoclonal antibodies or heat-inactivated sera were added to influenza virus, dosed at an amount of virus that resulted in 70% maximal NA activity. Subsequently, the serial dilutions were added to the wells of a 96-well plate that were coated with fetuin (25 μg/ml, Sigma Aldrich) and the plates were incubated for 16–18 hours at 37°C. Galactose residues that became exposed on the coated fetuin due to NA activity were detected with horse radish peroxidase (HRP)-coupled peanut agglutinin (PNA-HRP, 5 μg/ml, Sigma Aldrich).

### Hemagglutination inhibition (HI) assay

Hemagglutination and HI tests were performed in a round bottom 96-well microtiter plate at room temperature using 1% (vol/vol) chicken erythrocytes in PBS with 4 hemagglutinating units (HAU) of virus according to the WHO manual for influenza research [25].

### Statistical analysis

For comparison of two sets of values, a Student’s *t*-test (two-tailed, two sample equal variance) was used. When comparing three or more sets of values, the data were analyzed with One-way ANOVA followed by *post-hoc* analysis using Tukey’s multiple comparison test. Linear regression was used to assess correlations. All analysis was performed using GraphPad Prism software. *P* values of < 0.05 were considered significant.

## Results

### Antibodies directed against NA interfere with the early steps of influenza A virus infection of HAE cells

**Fig 1:**
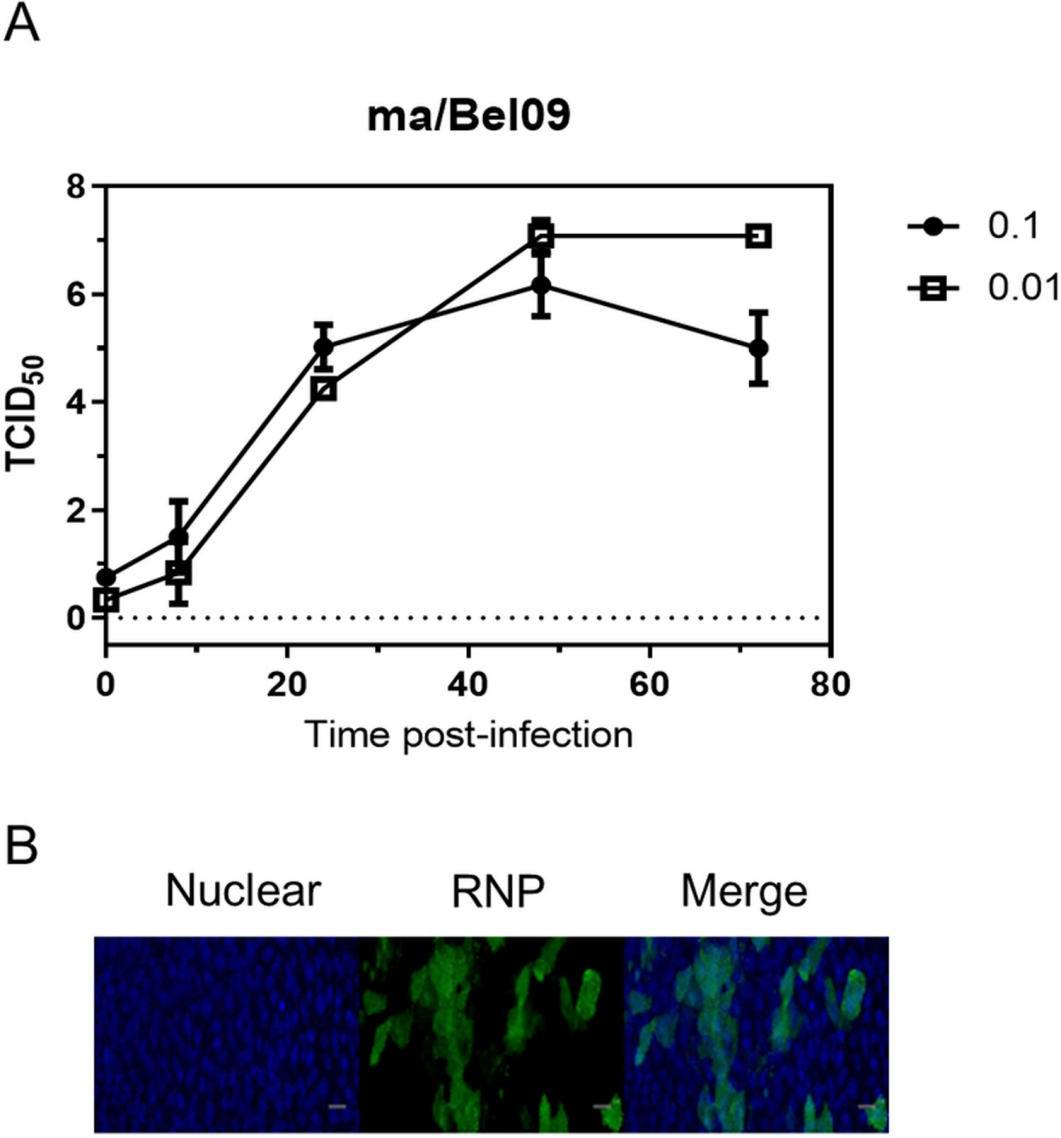
Infection of HAE cells by an A(H1N1)pdm09 virus. **(A)** Differentiated HAE cells (Lonza) were inoculated with a MOI of either 0.01 or 0.1 of maBel/09 for 1 h in triplicates. The inoculum was washed away and cells were maintained in the air-liquid phase. At 0, 8, 24, 48, and 72 hours post-infection, the apical side was sampled and virus was quantified by TCID_50_. Shown are the averages (±SD) of the TCID_50_ titers at each time point. **(B)** Differentiated HAE cells (Lonza) were infected with maBel/09 at a MOI of 1. The virus inoculum was removed after 1 hour of incubation. The cells were subsequently washed and 7 hours later fixed, permeabilised, and immune-stained for RNP. Hoechst was used to stain nuclei and cells were examined by widefield microscopy. Representative images taken with a 20x dry objective are shown in the bottom panel. Scale bar: 40 μm. Significance was assessed using two-way ANOVA.

Mucins that are present within the mucus layer that lines HAE cells can decrease the entry of influenza viruses into cells [2]. We first established the HAE infection model with a pandemic H1N1 2009 virus strain. This strain, maBel/09, used at a MOI of 0.01 and 0.1, could establish multi-cycle growth on the HAE cells, producing a peak virus release at 48 hours after inoculation (Fig 1A). Next, HAE cells were infected at a MOI of 1 with maBel/09 and we determined the presence of influenza virus ribonucleoprotein (RNP) positive cells at 8h after infection by immune-fluorescence. This time point after infection was chosen to restrict the analysis to the initial stages of infection (entry and start of viral transcription and replication) before a complete replication cycle was established. At 8 hours after inoculation, RNP-positive foci were clearly detectable in the HAE cells (Fig 1B).

**Fig 2:**
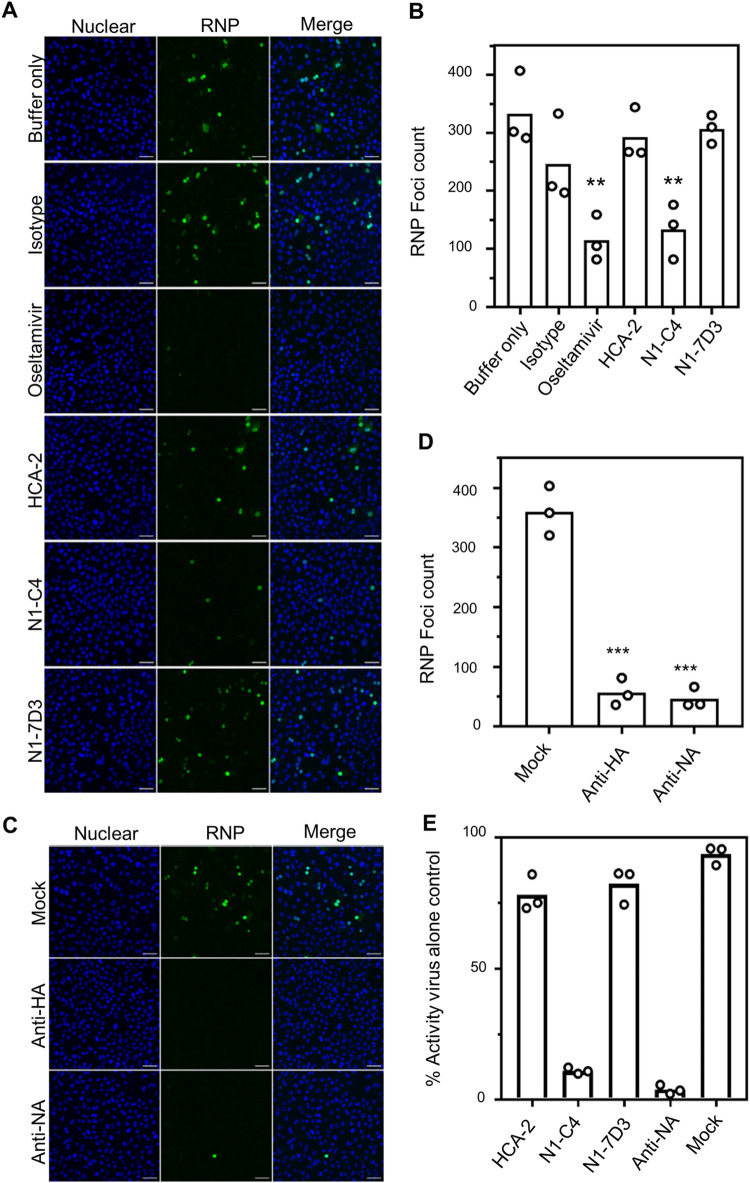
Antibodies with NI can restrict A(H1N1)pdm09 infection of HAE cells. **(A)** Differentiated HAE cells (Lonza), in triplicates, were inoculated with a MOI of 1 of maBel/09 that had been treated with either buffer alone, an IgG1 isotype control (10 μg/ml), Oseltamivir (24 μM), HCA-2 (10 μg/ml), N1-C4 (10 μg/ml), or N1-7D3 (10 μg/ml). The inoculum was washed away after 1h and 7h later the cells were fixed for immune-fluorescent imaging. Blue: DAPI; green: RNP. **(B)** quantification of the RNP foci from (A). **(C)** HAE cells (Lonza) were inoculated with maBel/09 virus that was preincubated with RDE-treated, and heat-inactivated mouse sera raised to PBS (mock), recombinant Bel/09 HA (anti-HA) or recombinant Bel/09 NA (anti-NA) at a 1:100 dilution. Eight hours after infection, the cells were fixed for immune-fluorescent imaging as in (A). **(D)** Quantification of the number of RNP foci from panel (C). Images in A and C display representative Zeiss 880 confocal microscopy images that were made using a 25x/0.8 numerical aperture multi-immersion objective. Scale bar: 40 μm. Image analysis was performed with Volocity (Perkin Elmer). HAE experiments were performed twice, shown are representative results from one experiment. (**E**) NA inhibition activity in anti-sera (heat inactivated) and the indicated monoclonal antibodies (10 μg/ml) determined by ELLA expressed as the percentage of the signal compared to the virus alone control. Bars represent the mean of the assay performed in triplicates where individual data points are overlayed. *p < 0.05, **p < 0.01, One-Way ANOVA in comparison to the isotype treated or mock controls.

To examine the possible impact of anti-NA antibodies on the early stages of infection, we tested a panel of NA-specific monoclonal antibodies. maBel/09 virus was pre-incubated with monoclonal antibody HCA-2 [29], N1-C4 [13], N1-7D3 [13], a mouse IgG1 isotype control monoclonal antibody, Oseltamivir, or buffer only. Preincubated viruses were then allowed to bind to the HAE cells for 1 hour, after which the inoculum was washed away and the cells were further incubated for 7 hours. The cells were subsequently fixed and the extent of infection was examined with a RNP-specific immune-fluorescent focus reduction assay. Incubation of the virus inoculum with N1-C4, which exhibits NI against maBel/09 virus, decreased viral infection compared to the buffer-only treatment (N1-C4 vs buffer only; p < 0.01 one-way ANOVA). Both HCA-2 and N1-7D3, which lacks NI activity, showed no significant reduction in viral replication compared to the untreated and isotype control treated maBel/09 (Fig 2A and 2B).

We next repeated the experiments using polyclonal mouse sera for virus pre-incubation, comparing immune sera from mice that had been immunized with recombinant soluble tetrameric NA or trimeric HA derived from the maBel/09, or from mice that had been immunized with adjuvant only. Both the NA and HA immune sera significantly reduced the number of RNP immune-reactive foci visualized and quantified at 8 hours after inoculation (anti-NA vs mock anti-serum; p < 0.001, one-way ANOVA, Fig 2C and 2D). To test if the ability of the antibodies and sera to interfere with the early stages of infection aligned with their ability to mediate NI, the antibodies and sera were tested in an ELLA at the same concentrations or dilutions as used for the HAE cells. N1-C4 and the anti-NA serum, but not HCA-2, N1-7D3, and the control immune serum, mediated NI against maBel/09 at the tested concentrations (Fig 2E).

**Fig 3:**
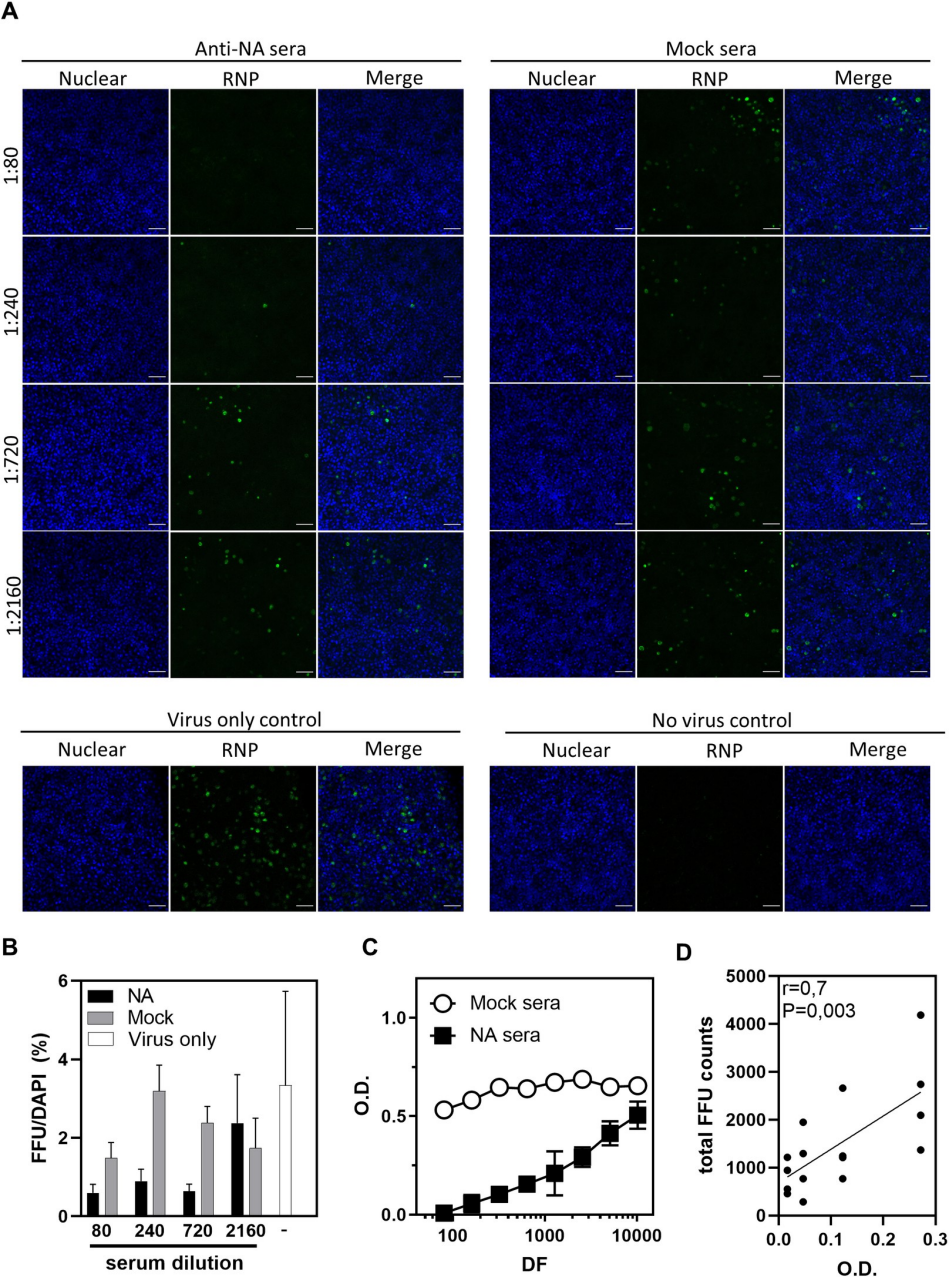
NI titers correlate with the ability of anti-NA sera to restrict infection in HAE cells. Serial dilutions of the RDE-treated and heat-inactivated sera raised in mice with adjuvanted PBS (mock) or recombinant Bel/09 NA (anti-NA) was used to treat maBel/09 prior to infection. Treated virus was added at a MOI of 1 to HAE cells (Epithelix) in quadruplicate and Bel/09 infection was assessed by a fluorescent focus reduction assay 8 h post-inoculation. **(A)** Randomly selected images taken with a Zeiss 880 confocal microscopy with 25x/0.8 numerical aperture multi-immersion objective. Scale bar: 40 μm. **(B)** Analysis of images was performed with Fiji (NIH) software and represented here as the percentage of positive focus forming units (FFU) per DAPI counts. **(C)** NI of the mock and anti-NA sera. Heat-inactivated only sera were mixed with a pre-determined amount of Bel/09 and added to fetuin coated wells. After 18 h, the amount of exposed galactose residues was determined with PNA-HRP in an ELLA. The assay was performed in triplicate and is representative of 2 independent experiments. **(D)** Linear regression analysis between the total number of foci and the optical densities (O.D.) measured in the ELLA and Spearman’s rank correlation coefficient. Each dot represents the total foci counts per HAE membrane (n = 4 per serum dilution).

We next performed a dose titration experiment to try to correlate the requirement for NI activity for the suppression of the early stages of maBel/09 infection of HAE cells. Anti-Bel/09 NA or control immune serum was diluted to 1:80, 1:240, 1:720, and 1:2160 before pre-incubation with maBel/09 virus that was used for inoculation. At a 1:80 to 1:720 dilutions, but not at higher dilutions, the anti-NA serum suppressed virus infection (Fig 3A and 3B). A significant correlation was observed between the optical density of the fetuin/PNA-HRP-based ELLA assay, in which a low optical density reflects low NA activity (thus high NI activity), and the total number of foci (P = 0.003, r^2^ = 0.7, Spearman correlation) (Fig 3C). In summary, the data suggest that monoclonal antibodies and polyclonal mouse sera with NI activity can restrict influenza A virus entry in HAE cell cultures.

### Antibodies towards NA can decrease replication over-time and restrict egress from the cell surface

**Fig 4:**
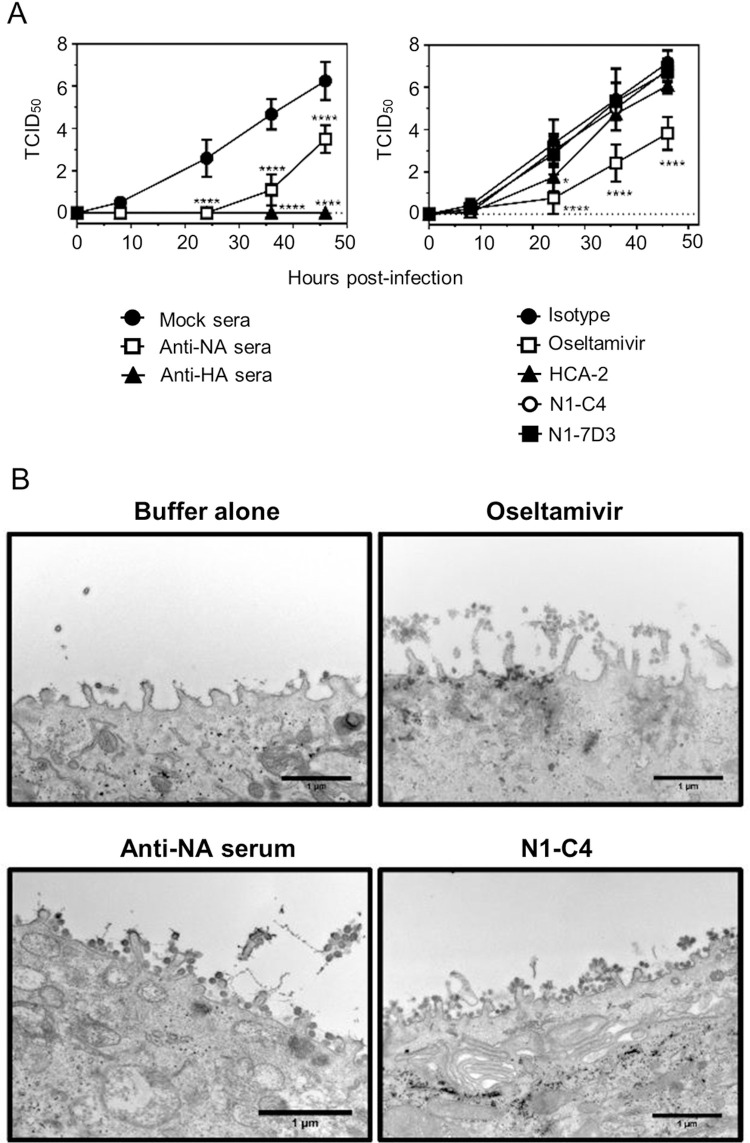
N1-specific anti-serum decreases replication of A(H1N1)pdm09 in HAE cells. **(A)** maBel/09 at a MOI of 0.01 was pre-treated with (i) mock serum, (ii) anti-NA serum, (iii) anti-HA serum, (iv) IgG1 isotype control, (v) Oseltamivir, (vi) monoclonal antibody HCA-2, (vii) N1-C4, or (viii) N1-7D3 and added to HAE cells (Lonza) for 1 h. All sera were RDE treated and heat-inactivated. The sera were used at 1:100 dilution, monoclonal antibodies at 10 μg/ml, and Oseltamivir at 24 μM. The inoculum was washed away and anti-sera, monoclonal antibodies, or Oseltamivir were re-added. At 0, 8, 24, 36, and 46 h post inoculation, the presence of virus released at the air-liquid interface was determined by TCID_50_. The average viral titers from triplicate HAE cultures ± SD is shown. The data is representative of two independent experiments. *p < 0.05, ***p < 0.001, ****p<0.0001 two-way ANOVA in comparison to either mock sera or isotype control. **(B)** NA-specific polyclonal sera and N1-C4 restrict virus egress from the surface of HAE cells. maBel/09 at a MOI of 1 was incubated with buffer alone, Oseltamivir (24 μM), anti-NA serum (1:100 final concentration), or N1-C4 (10 μg/ml final concentration) and added to washed HAE cells (Lonza). The virus was allowed to bind for 1 h, after which the inoculum was removed by washing. Buffer, Oseltamivir, anti-NA serum, or N1-C4 was re-added for a further 24 h. The cells were fixed and processed for TEM. Shown are representative images taken with a JEM1010 TEM microscope at 10,000x magnification. Scale bar: 1 μm.

We next examined the ability of polyclonal anti-NA sera and monoclonal antibodies to alter infection over time. Anti-NA, anti-HA, or mock control immune serum (1:100 final dilution) and NA-specific monoclonal antibodies or irrelevant control monoclonal antibodies (10 μg/ml final concentration), and Oseltamivir were pre-mixed with maBel/09 virus. After one hour of incubation on HAE cells, the inoculum applied at a MOI of 0.01 was removed, and the cells were incubated in the air-liquid phase. After the initial inoculum wash and after each sampling step, antibodies were re-added to maintain a constant concentration of antibodies at the air-liquid interface throughout the experiment. Anti-NA serum significantly delayed the production of new virus in comparison to mock serum-treated maBel/09 (Fig 4A, left panel). In contrast, NA-specific monoclonal antibodies, at 10 μg/ml, did not significantly affect viral replication over time, except for HCA-2, which resulted in slightly reduced maBel/09 replication compared to the isotype control at 24 hours (Fig 4A, right panel).

NA-specific antibodies with NI activity could also interfere with the release of nascent viruses. For Oseltamivir this has been clearly demonstrated in MDCK cells [30] and there is evidence that polyclonal NA anti-sera can hamper the release of H2N2 virus from chicken embryonic fibroblasts [31]. To further examine if anti-NA antibodies could suppress viral egress from HAE cells, TEM was used to image the cell surface 24 hours post infection with maBel/09 (MOI 1) in the presence of buffer alone, Oseltamivir, anti-NA serum, or N1-C4. For the buffer alone control little virus could be observed at the surface of the cells. In contrast, EM photomicrographs of Oseltamivir-, anti-NA serum-, and N1-C4-treated and infected HAE cells, revealed clusters of A(H1N1)pdm virions on the plasma membrane surface, indicating that these treatments restricted newly produced virus egress from the infected cell surface (Fig 4B).

### Human sera with Bel/09 NA NI activity restrict the early stage of H6N1 infection of human airway epithelial cells

**Fig 5:**
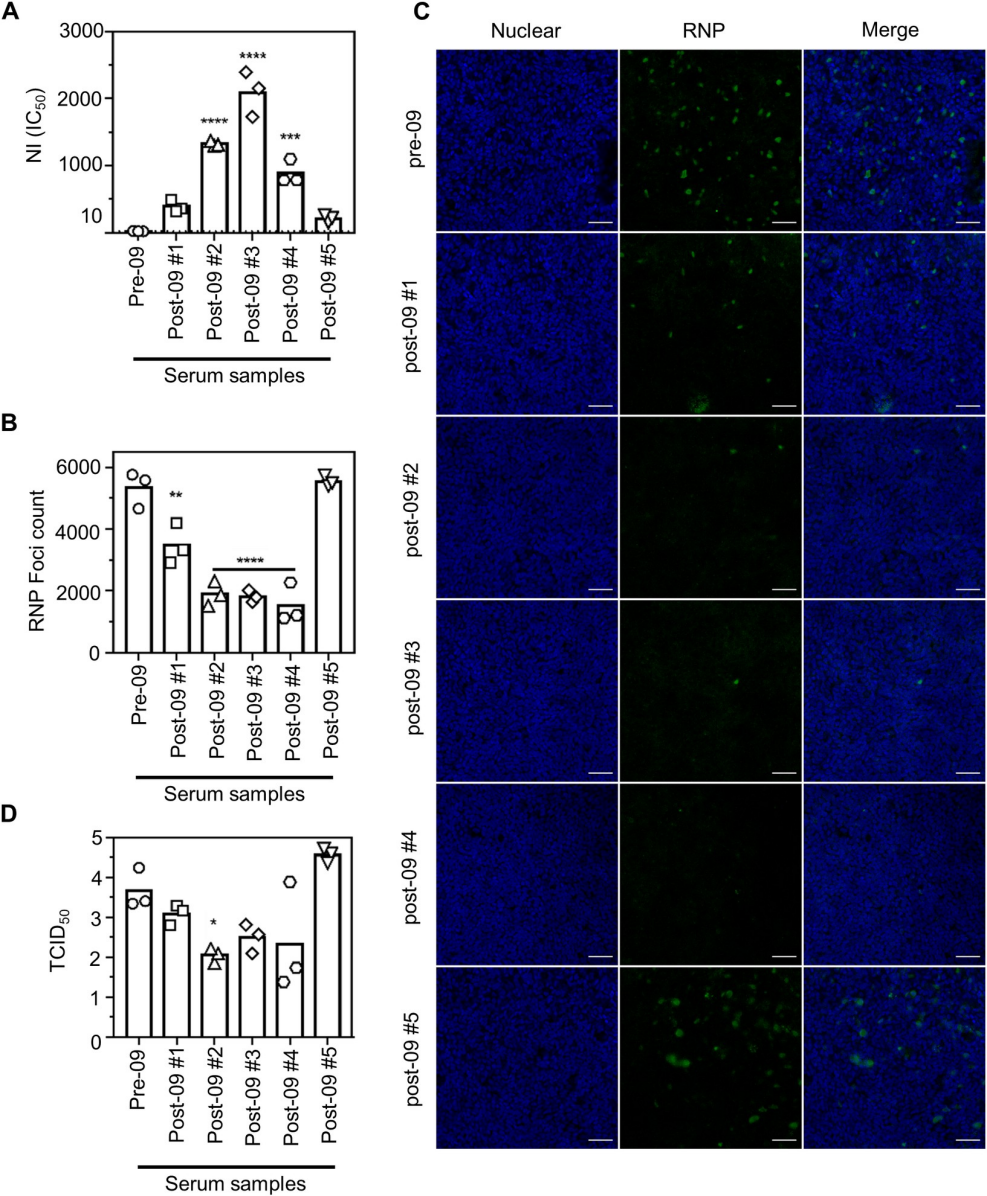
Human sera restrict growth of H6N1 virus in HAE cells. **(A)** Heat-inactivated human serum samples were tested for NI in an ELLA alongside a pre-2009 human serum sample. Shown is the IC_50_ titer of the ELLA performed in triplicate and is representative of two independent experiments. Bars represent the average of the triplicates which are overlaid. 1:20, the lowest dilution tested, represents the cut-off of the assay. **(B-D)** Human sera with NI activity control H6N1 replication in HAE cells. HAE cells (Epithelix) were inoculated with a MOI of 0.1 of RG H6N1_Bel/09_ that had been pre-treated with (i) a human pre-2009 serum sample or (ii) human sera obtained one week after administration of a quadrivalent 2017–2018 season vaccine. One hour after infection the inoculum was washed away and 23 hours later, the apical side was sampled for virus titration and the HAE cells subsequently fixed, permeabilised and examined for replication. Sera used was heat-inactivated and RDE-treated at a final dilution of 1:40. The assay was performed in triplicate. **(B)** Average bar graphs with individual triplicate points overlayed from the sum of vRNP-positive foci counted from the maximum intensity projections from each membrane. Image analysis was performed with Volocity (Perkin Elmer). **(C)** Representative fluorescent microscopy images of HAE cells grown on a membrane taken by a Zeiss 880 confocal microscopy with the 25x/0.8 numerical aperture multi-immersion objective. Scale bar: 50 μm. **(D)** Virus titer sampled from the apical side of the HAE cells at 24 h post-inoculation determined by TCID_50_ *p < 0.05, **p < 0.01, ***p < 0.001, ****p<0.0001, one-way ANOVA in comparison to pre-2009 serum.

We next wondered whether human serum with NI antibodies could also interfere with entry of influenza virus into HAE cells. To address this, sera were obtained from healthy volunteers one week after they had received a quadrivalent 2017–2018 Northern hemisphere influenza vaccine. Five sera were selected based on their ability to bind to purified recombinant Bel/09 NA in ELISA (S2 Fig) to assess their ability to inhibit NA activity of H6N1_Bel/09_ virus. This virus carries the NA segment of Bel/09 virus, the HA segment of A/mallard/Sweden/91/2001 and the other 6 segments from PR8. The H6 subtype has not circulated within the human population and, consistent with this, no HI titers could be detected against the H6N1_Bel/09_ virus with any of the five human sera (HAI titer < 20). Therefore, in a fetuin-based NI assay, the NI response against NA of the H6N1 virus could be examined without interference from HA-head specific antibodies. Human serum sourced prior to the 2009 H1N1 pandemic displayed no meaningful ability to inhibit NA activity of the H6N1_Bel/09_ virus. The 5 other selected human sera could inhibit NA to varying levels (Fig 5A). Post-09 sera #2, #3, and #4 showed significant IC_50_ titers compared to Pre-09 serum, whereas sera #1 and #5 showed lower IC_50_ titers which were not significantly different from the Pre-09 serum (Fig 5A).

We then used these human sera to examine their potential to inhibit H6N1_Bel/09_ virus entry into HAE cells. H6N1_Bel/09_ virus at a MOI of 0.1 was pre-incubated with a 1:40 dilution of the human sera and foci were quantified 24 hours post-infection. Sampling from the apical side was also performed to determine the amount of newly produced H6N1_Bel/09_ virus at 24 hours post infection. Human post-vaccination serum samples #1, #2, #3, and #4 significantly reduced the number of H6N1_Bel/09_ foci compared to pre-2009 human control serum, whereas serum #5 did not (Fig 5B and 5C). In addition, human sera #2, #3, and #4 showed a modest reduction on newly produced virus, sampled from the apical side of the HAE cultures, which was significantly different from the pre-09 treated serum sample for serum sample #2 (p < 0.05, one way ANOVA). Together this data suggests that human sera with strong NI activity can reduce influenza A virus replication in differentiated primary airway epithelial cell cultures.

## Discussion

Compared to HA-directed antibodies, the possible mechanisms of protection mediated by anti-NA antibodies are less well defined. Studies by Sakai *et al*. [4] and Guo *et al*. [5] revealed the need for NA in the influenza virus entry process, whereby NA in cooperation with HA drives motility of the virus on the cell surface. The authors of these two studies speculated that this process may allow the virus to reach a suitable endocytic patch that can trigger virion internalization. Additional reports have also implicated NA in the HA mediated fusion process [6, 32]. The addition of small molecule NA inhibitors has been shown to interfere in both of these processes and decrease infection levels in cells [4, 33]. Here we report that antibodies with NI activity can interfere with the initial round of infection in HAE cells. Whether NI antibodies prevent or delay access to receptors on the cell surface, endocytosis and/or the fusion process is not yet known and warrants further investigation. Unlike HA head-specific antibodies, which can neutralize influenza viruses *in vitro*, infection of HAE cells in the presence of NI antibodies is not completely prevented, since some RNP positive cells could be detected after 8 hours, albeit significantly lower number of cells than in control treated samples. However, after multiple rounds of infection a restrictive effect of this early onset delay was no longer detectable when anti-NA antibodies were not continuously present. When the anti-NA antibody concentrations were maintained throughout the HAE infection process, however, and provided that a certain threshold of NI activity was present in the serum, infection of H1N1 maBel/09 and H6N1_Bel/09_ virus was significantly delayed. It would be expected that other NA inhibitory mAbs such as 1G01 or CD6 would also be able to reduce H1N1 virus infection of HAE cells [34, 35].

Human sera with NI antibodies reduced H6N1_Bel/09_ virus replication into HAE cells. The use of the H6N1-reverse genetics virus allowed to circumvent the interference of antibodies directed to HA of H1N1 and H3N2 viruses that are present in the serum of adults, in the ELLA assay. However, we cannot rule out the possibility that HA stalk-specific antibodies contributed to some extent to the inhibition observed in ELLA or the decreased H6N1 virus entry observed in HAE cells. Some stalk antibodies have been shown to interfere with NA activity in ELLAs [36] and to also block the IAV fusion process, which suppresses replication [37, 38].

The currently licensed inactivated influenza vaccines do not contain a standardized level of NA and induce reduced anti-NA responses compared to natural infections [39, 40]. Moreover, there is evidence that serum anti-NA responses can persist for multiple seasons following vaccination of healthy volunteers with inactivated or live attenuated influenza vaccines [41]. Of further interest would be to address if serum samples from recipients of high dose Fluzone®, often administered to the elderly population and reported to induce higher levels of NI than standard dose inactivated influenza vaccine, may induce more robust suppression of influenza virus replication in the HAE cells [42].

The presented TEM data showed the ability of N1-C4, Oseltamivir and polyclonal anti-NA serum to restrict virus egress from the cell surface (Fig 4B). However, for N1-C4, the ability to restrict virus release was not reflected in the virus sampled from the apical side of the culture in the multistep growth curve. There may be multiple reasons for this observation. Firstly, the polyclonal anti-NA serum displayed stronger NI activity than N1-C4, at the dilutions, respectively, concentrations tested. Therefore, the effect of the anti-sera across multiple rounds of infection would be stronger, resulting in significantly reduced viral titers. Secondly, the TEM assessment is qualitative, not quantitative, and we imaged only a single time point after infection. Patches of viral egress inhibition may have been sparser in the N1-C4 setting compared to polyclonal anti-NA serum. Finally, it cannot be ruled out that the method of sampling at the apical side could release virus trapped at the surface of the cell in the glycocalyx resulting in higher viral titers in the TCID_50_ read-out.

The studies presented here focused on the A(H1N1)pdm09 virus. Future studies are needed to confirm whether antibodies directed to other NA subtypes can also control infection in this manner. Matrosovich *et al*. reported that Oseltamivir could indeed inhibit the entry of various subtypes of IAV into HAE or primary human nasal epithelial cells including seasonal H1N1s, H7N1, and H7N7 viruses of avian origin and the A/Sydney/5/1997-like H3N2 strain [3]. The HA of modern H3N2 viruses displays a decreasing affinity for sialic acid while the NA appears to be compensating for this, displaying a higher affinity for sialic acid than in the past and mediating entry into cells even in the presence of a HA that fails to bind sialic acid (reviewed in [43]). It would be of interest to see if NI antibodies against contemporary H3N2 viruses could also decrease the initial entry into cells. A N-glycosylation at position 245 near the catalytic site of recent N2 NAs has been reported to confer partial resistance to inhibition by sera raised against NA that do not carry a N-glycan at this position [44]. N2 NA N-glycosylation also affects the binding of mAbs HCA2, 235-1C02 and 229-1G03 [45]. It is therefore expected that H3N2 viruses that have acquired an N-glycosylation at position 245 would be partially resistant to inhibition by immune sera raised against NA derived from previously circulating H3N2 viruses that lack this N-glycosylation site. Moreover, targeting NA may increase the efficacy of next generation influenza vaccines, as antibodies against NA could help aid in the control of viruses which, for receptor binding, are becoming less-dependent on the HA, the primary target of current vaccine strategies.

## Supporting information

S1 Fig Glycan rich composition is maintained on HAE cells after wash with PBS. A549 or HAE cells washed 3x with PBS or non washed were stained with Periodic Acid-Schiff.
(PPTX)

S2 Fig Anti-NA serum IgG titers. ELISA was performed using tetrabrachion stabilized NA with sera from human donors. The end point titer was determined for each serum sample by scoring the dilution that had an O.D. that was equal to or higher than two times the background O.D. obtained from the control sera (naïve mice) dilution series.
(PPTX)
