# Supplementary material for: Antibodies directed towards neuraminidase restrict influenza virus replication in primary human bronchial epithelial cells
Source: PLoS One. 2022 Jan 31;17(1):e0262873. doi: 10.1371/journal.pone.0262873 (PMC8803191; doi:10.1371/journal.pone.0262873)
Supplement: S1 Fig — A549 or HAE cells washed 3x with PBS or non washed were stained with Periodic Acid-Schiff. (PPTX) [file pone.0262873.s001.pptx]

## Slide 1
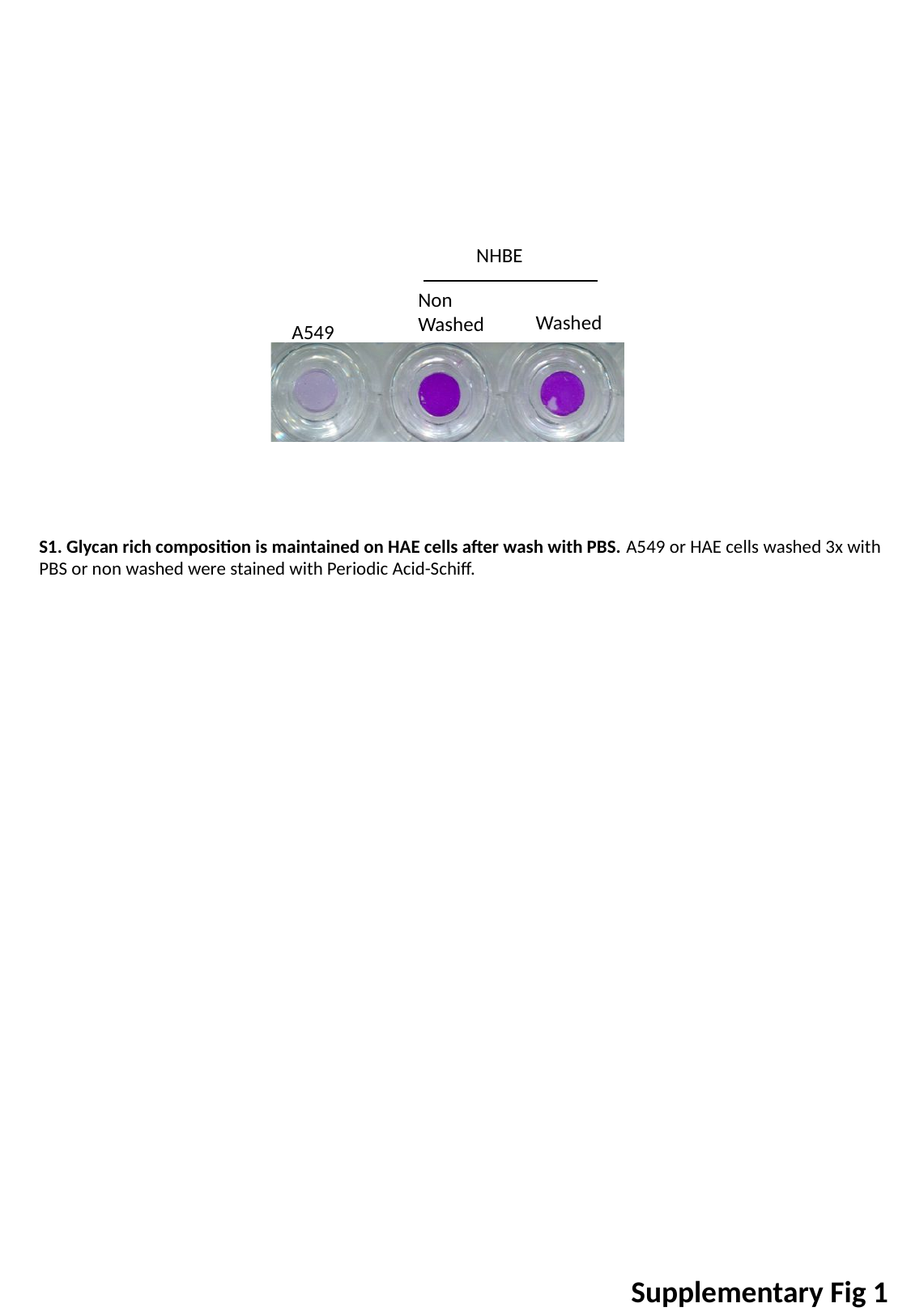

NHBE
A549
Washed
Non
Washed
S1. Glycan rich composition is maintained on HAE cells after wash with PBS. A549 or HAE cells washed 3x with PBS or non washed were stained with Periodic Acid-Schiff.
Supplementary Fig 1
