# Supplementary material for: Antibodies directed towards neuraminidase restrict influenza virus replication in primary human bronchial epithelial cells
Source: PLoS One. 2022 Jan 31;17(1):e0262873. doi: 10.1371/journal.pone.0262873 (PMC8803191; doi:10.1371/journal.pone.0262873)
Supplement: S2 Fig — ELISA was performed using tetrabrachion stabilized NA with sera from human donors. The end point titer was determined for each serum sample by scoring the dilution that had an O.D. that was equal to or higher than two times the background O.D. obtained from the control sera (naïve mice) dilution series. (PPTX) [file pone.0262873.s002.pptx]

## Slide 1
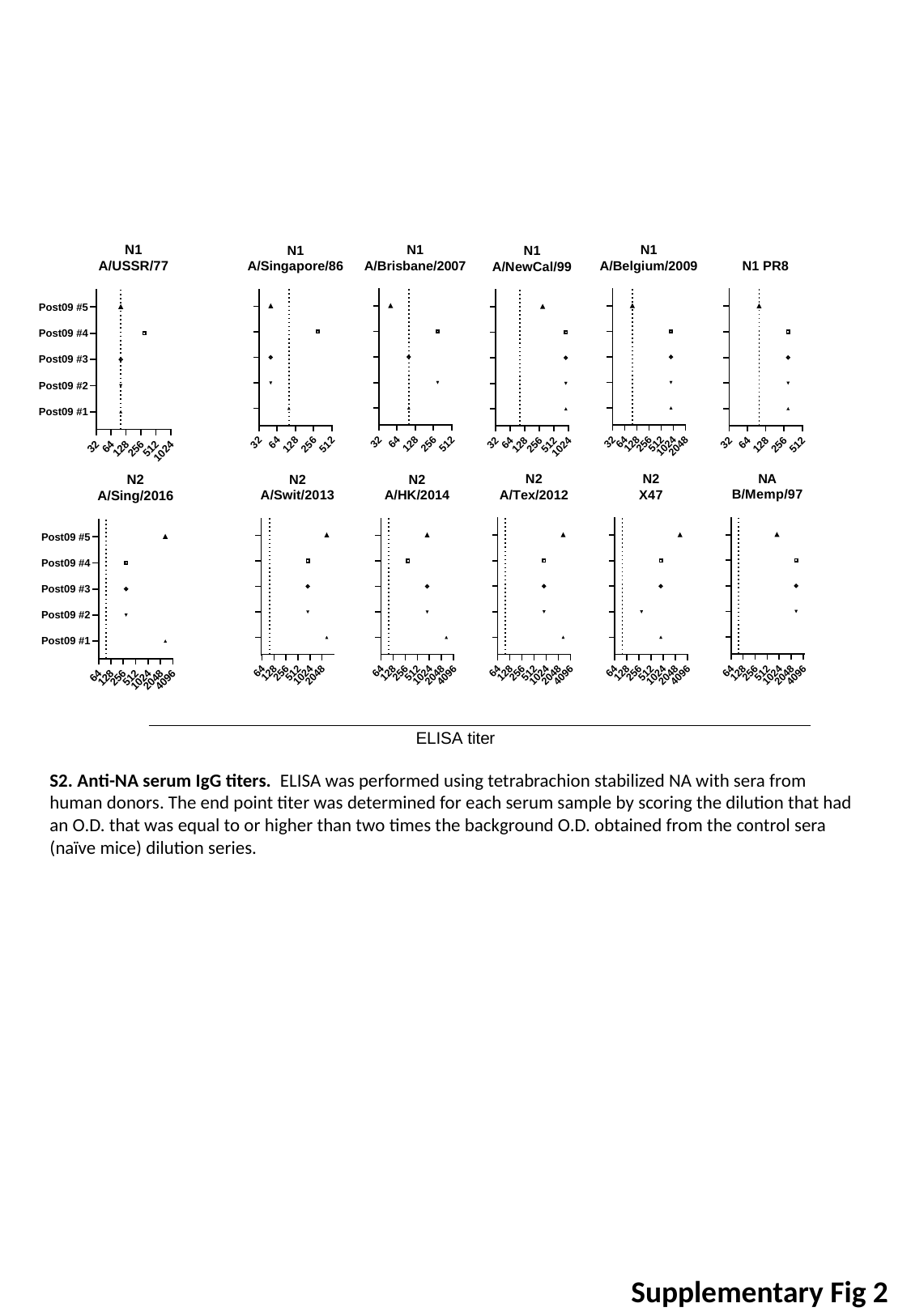

S2. Anti-NA serum IgG titers. ELISA was performed using tetrabrachion stabilized NA with sera from human donors. The end point titer was determined for each serum sample by scoring the dilution that had an O.D. that was equal to or higher than two times the background O.D. obtained from the control sera (naïve mice) dilution series.
Supplementary Fig 2
